# Primary localized laryngeal amyloidosis presenting with hoarseness and dysphagia: a case report

**DOI:** 10.4076/1752-1947-3-9049

**Published:** 2009-09-16

**Authors:** Ioannis Yiotakis, Alexandros Georgolios, Alexandros Charalabopoulos, Panagiotis Hatzipantelis, Christos Golias, Konstantinos Charalabopoulos, Leonidas Manolopoulos

**Affiliations:** 1 Department of Otorhinolaryngology, Medical Faculty, University of Athens, Hippokration General Hospital, Athens, Greece; 2 Department of Physiology, Clinical Unit, Medical Faculty, University of Ioannina, Ioannina, Greece; 3 Department of Pathology, Hippokration General Hospital, Athens, Greece

## Abstract

**Introduction:**

Primary localized laryngeal amyloidosis is an extremely rare condition. It usually presents with hoarseness, pain and/or difficulty in breathing.

**Case presentation:**

We present the case of a 23-year-old woman with primary localized laryngeal amyloidosis who presented with hoarseness and dysphagia.

**Conclusion:**

A search of PubMed shows that dysphagia in patients with laryngeal amyloidosis has been reported only once, although this symptom is relatively common in other conditions presenting with laryngeal mass. There were no signs of any systemic disease in our patient and diagnosis was established histopathologically. She was treated surgically by microlaryngoscopy under general anesthesia and the mass was excised using a CO_2_ laser technology method.

## Introduction

Amyloidosis is a benign, slowly progressive condition that is characterized by the presence of extracellular fibrillar proteins in a variety of organs and tissues. Localized deposition of amyloid may be observed in individual organs without any systemic involvement. The progressive accumulation of amyloid deposits interferes with the normal structure of affected tissues resulting eventually in impairment of their function. Localized deposition of amyloid protein is regarded to be the result of local synthesis rather than the deposition of light chains produced elsewhere in the human body.

Primary laryngeal amyloidosis is a rare lesion representing 1% of all benign laryngeal tumors [1]. Dozens of cases of head and neck organ amyloidosis have been reported in the literature since Borow [2] described the first case in 1873. Immunohistochemical stains, and Congo red staining viewed under polarized light microscopy, or electron microscopic findings of a laryngeal biopsy specimen can confirm the presence of amyloid in the larynx. A high degree of suspicion is necessary since clinical presentation of the disease may mimic that of a laryngeal neoplasm. Therefore, there is a high risk if the clinician takes the wrong diagnostic approach. We recommend that an adequate deep punch biopsy must be obtained in order to exclude malignancy. Furthermore, an experienced pathologist should examine the lesion histologically with routine stains (stain pink with hematoxylin-eosin stain and show metachromasia with crystal violet) mainly to exclude malignancy. However, tissue specimens should also be stained with Congo red and examined under polarized light microscopy to establish the diagnosis of the disease.

We present a case that underlines the role of birefringence from red Congo stain to confirm the diagnosis. Another important feature of our case was that dysphagia was the main presenting symptom.

## Case presentation

A 23-year-old Caucasian Greek woman presented with a history of progressively worsening hoarseness during the previous 9 months and recent onset of dysphagia. She reported no weight loss and she denied the use of tobacco or excessive alcohol consumption. She had a history of skin atopy, reporting sensitivity to wool, and a history of an episode of anaphylactic reaction of unknown cause 2 years previously.

**Figure 1:**
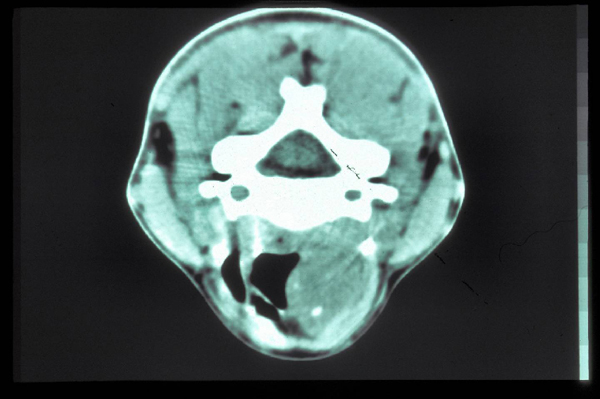
**Computed tomography scan at the level of the supraglottis**.

Physical examination of the neck was normal. Indirect laryngoscopy and endoscopy with a flexible endoscope revealed a smooth, red-yellow, cystic formation localized in the left hemilarynx between the false vocal cord and the aryepiglottic fold. Chest X-ray was normal. A tracheal computed tomography (CT) scan in 5 mm sections confirmed the existence of the mass (Figure 1). The thyroid cartilage appeared intact and no nodal involvement was detected.

We proceeded to perform microlaryngoscopy under general anaesthesia and removal of the mass (measuring 4 × 2.5 × 2 cm) using a CO_2_ laser. Pathological examination revealed a mass with a smooth surface. On macroscopic inspection the cut sections were yellow-grey and solid with a soft and elastic consistency. On microscopy, it was not clear whether its structure was bundled or micronodular, consisting of bundles of woven eosinophilic tissue, with sparse fibroblastic cells, several small vessels and rare atrophic glandular regions. There were also a few collections of lymphocytes and granulocytes. Staining with Congo red stain, under polarized light, revealed blue-green birefringence throughout the mass due to the presence of amyloid. No signs of malignancy were seen.

Further examinations were done to rule out systemic amyloidosis. The patient's complete blood count, erythrocyte sedimentation rate, basic metabolic and biochemical panel and liver function tests were within normal limits. Serum calcium was also normal. Serum and urine electrophoresis were normal. Rectal biopsy was negative. Based on these findings systemic amyloidosis and multiple myeloma were excluded from the differential diagnosis. The amyloid light chain was lambda (λ) type. The amyloid mass was removed by microlaryngoscopy.

## Discussion

Amyloidosis is a metabolic protein disturbance in which extracellular protein fibrils are deposited in various tissues. Amyloidosis is classified as systemic or local. According to the type of amyloid, classification of amyloidosis appears as follows for systemic and localized amyloidoses respectively: hereditary amyloidosis, for example, amyloidosis in familial Mediterranean fever with types AA, AF amyloid; idiopathic systemic amyloidosis with AL amyloid; secondary systemic amyloidoses (reactive amyloidosis) and chronic infections, neoplastic diseases with AA amyloid and localized amyloidoses with AL, AA, AK types of amyloid. Table 1 summarizes the classification of amyloidosis with the accompanying amyloid type. Localized amyloidosis occurs in a variety of organ systems. Both types may affect the upper and lower respiratory tracts. In the past, the extracellular deposition of amyloid in the larynx was commonly misdiagnosed as vocal cord nodules. It is now certain that the latter are due to deposition of fibrin and other factors of local blood exudation and are unrelated to the rare amyloidosis [1,3]. Primary lesions located in the larynx were first reported in 1873; however, the real pathogenesis of the entity has not been fully elucidated until now. Apart from a case of familial primary localized laryngeal amyloidosis in two sisters and primary localized amyloidosis in various human organs, familial primary localized amyloidosis of the larynx has not yet been reported [4]. The familial type of the disease (ATTP - type amyloid transthyretin protein) is extraordinarily rare and exhibits an autosomal dominant pattern of inheritance. The extracellular deposits of amyloid may be focal, limited in one tissue or organ, or may present systemic distribution. They rarely show remission but usually tend to increase in size at a slow but progressive rate. The larynx is rarely the first location of systemic amyloidosis; however, the latter should not be ruled out from the differential diagnosis because of its potentially ominous prognosis.

**Table 1:** Classification of amyloidosis

	Type of amyloid
Systemic amyloidoses	
I. Hereditary amyloidosis (e.g., amyloidosis in familiar Mediterranean fever)	AA, AF
II. Idiopathic systemic amyloidosis	AL
III. Secondary systemic amyloidoses (reactive amyloidoses) and chronic infections, neoplastic diseases	AA
Localized amyloidoses	AL, AA, AK

The ventricles and the false and true vocal cords are the most common sites for localized amyloidosis in the respiratory tree [5]. Other sites are the eye, the orbits and the major and minor salivary glands, while submucosal deposits have been observed in the nose, paranasal cavities, nasopharynx, oral cavity, stomatopharynx, bronchotracheal tree and lungs [6]. Oral and paranasal amyloidosis is usually a manifestation of systematic amyloidosis, mainly plasma cell dyscrasia [7]. Laryngeal amyloidosis usually appears during the fifth and/or sixth decade of life, without specific symptoms, though it most frequently involves hoarseness of the voice [1]. Dyspnoea is a common manifestation of the disease. Interestingly, apart from hoarseness, our patient was still relatively young to be complaining of dysphagia. A PubMed search shows that dysphagia as a clinical symptom has been reported only once [7]. Additionally, the youngest cases reported so far in the literature were those of an 11-year-old girl and a 12-year-old girl [8,9].

Regarding the requested laboratory control, in cases of localized laryngeal amyloidosis, Lewis *et al*. [10] studied 22 patients in the Mayo Clinic suffering from amyloidosis located exclusively in the larynx during the period 1950 to 1988 and recommended urine and serum electrophoresis as a basic initial approach. They did not recommend bowel and bone marrow biopsies as absolutely necessary.

The most commonly used method to detect the amyloid protein is the histological staining of biopsy samples excised with Congo red stain. Amyloid is birefringent in polarized light and appears apple-green in color (the so-called dichroism) in Congo red stained sections. This should be distinguished from pseudoamyloid, which is often found in vocal cord nodules. This is of fibrous consistency and represents an amorphous granular degeneration of collagen fibers with sparse disseminations between the fibroblasts [11]. Potassium permanganate may be used for the discrimination of protein composition between type A protein (AA) which dissolves, and amyloid (AL) of light chain, which is resistant and appears in the sections. Laryngeal amyloidosis is a type of localized amyloidosis that is characterized by monoclonal deposits of the light chain type (AL) [10].

Magnetic resonance imaging (MRI) is the technique of choice to detect the most specific features, since amyloid deposits present an intermediate T1-weighted signal intensity and low T2-weighted signal intensity, and MRI is thus considered to be a more specific technique than CT scanning [12]. Unfortunately, in our case, it was not possible to conduct an MRI scan due to technical reasons (the university hospital magnet was out of order). Thus, regarding the diagnostic approach, a high disease suspicion index followed by serum and urine electrophoresis, rectal biopsy, punch biopsy during direct laryngoscopy and MRI constitute an effective diagnostic procedure. Of course, the pathologist—as mentioned above—contributes significantly in the diagnosis of the disease.

**Figure 2:**
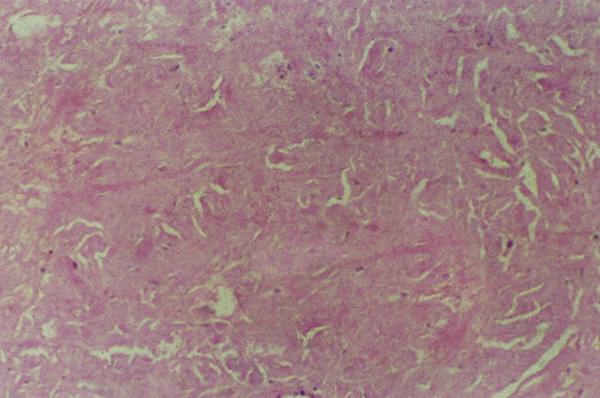
**Aggregates of acellular eosinophic material typical of amyloid associated with sparse inflammatory cells (H&E × 100)**.

**Figure 3:**
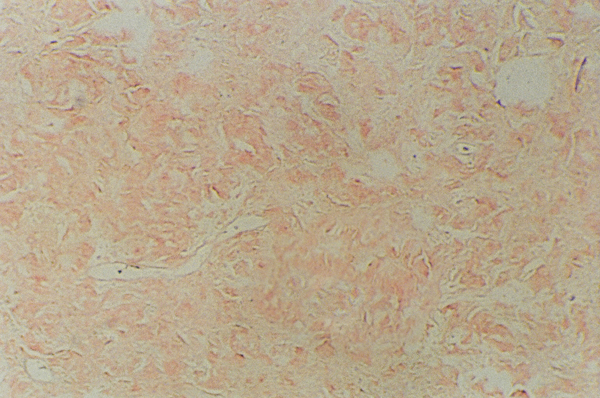
**The aggregates of amyloid stain with Congo red**.

In our case, the excised mass was oval shaped, uncapsulated, tumor-like yellow-gray nodule. Microscopically, the mass was composed of micronodules consisting of amorphous, acellular, eosinophilic, glassy material with variable inflammatory reactions including lymphocytes (Figure 2). Foreign body giant cells were also seen engulfing fragments of amyloid. Congo red with associated apple-green birefringence under polarized light was diagnostic of amyloid (Figure 3).

There are indications that immunological mechanisms are involved in the pathogenesis of human amyloidosis and that the latter could be a complication in immunodeficient conditions. Factors that affect the human immune response, such as steroids, immunosuppressive and ionizing radiation, may accelerate the appearance of the disease. Some types of amyloid deposits may be the result of an immunocyte dyscrasia or mucosa associated lymphoid tissue neoplasms [13]. Although systemic amyloidosis presents a poor prognosis due to accumulation of amyloid protein in a variety of vital organs impairing their structure and function, localized primary amyloidosis carries a much better prognosis. Therefore, it is crucial to identify the local presence of the amyloid protein by the above-mentioned procedures, since in contrast to systemic amyloidosis, local amyloidosis presents a very good prognosis. The treatment of primary localized laryngeal amyloidosis is surgical and may be performed with the aid of laser technology. Endoscopic CO_2_ laser excision of the mass should be the first line of therapy. The course of the condition under discussion is slow but sudden relapse is possible [14]. However, relapse may occur after a long time period; long-term follow up is essential for at least 5 to 7 years [1]. In this case, evolution into the systemic form of the disease was not observed in a 20-month follow-up; there was no disease recurrence and the patient was free of symptoms in this time period and was in a good state of health. It is important to mention that in contrast to local amyloidosis which carries a much better prognosis, evolution into the systemic form of the disease carries a poor prognosis because the accumulation of amyloid fibrils in the tissues interferes with their normal structure and function. In the study by Biewend *et al*. [1] in a mean 7.6-year follow up of two patients no recurrence was observed, while in another study by Piazza *et al*. [15], 17 out of 32 patients were asymptomatic in a 20-year follow up. In secondary amyloidosis, a reduction in amyloid deposition may occur following successful treatment of the underlying disease.

## Conclusions

Our case report is the second one in which dysphagia is referred to as a disease symptom in primary localized amyloidosis. The diagnosis of the disease is always established histologically; surgical excision of the mass by microlaryngoscopy using a CO_2_ laser technology method was the therapy of choice.

## Consent

Written informed consent was obtained from the patient for publication of this case report and any accompanying images. A copy of the written consent is available for review by the Editor-in-Chief of this journal.

## Competing interests

The authors declare that they have no competing interests.

## Authors' contributions

IY, LM, AG and AC analyzed the patient's medical data regarding the disease and performed the treatment. They were also involved in drafting the manuscript, making substantial contributions to the conception and design of the manuscript. CG and KC analyzed and interpreted the patient's clinical and laboratory data and were major contributors in writing the manuscript and revising it critically for important intellectual content. PH performed the histological examination of the mass and interpreted the results. He also contributed in drafting the manuscript. All authors read and approved the final manuscript.
